# Regulation of Plant Immunity through Modulation of Phytoalexin Synthesis

**DOI:** 10.3390/molecules19067480

**Published:** 2014-06-06

**Authors:** Olga V. Zernova, Anatoli V. Lygin, Michelle L. Pawlowski, Curtis B. Hill, Glen L. Hartman, Jack M. Widholm, Vera V. Lozovaya

**Affiliations:** 1 Department of Crop Sciences, University of Illinois, 1201 W. Gregory Drive, Urbana, IL 61801, USA; E-Mails: zernova@illinois.edu (O.V.Z.); lygin@illinois.edu (A.V.L.); mpawlow4@gmail.com (M.L.P.); curthill@illinois.edu (C.B.H.); widholm@illinois.edu (J.M.W.); 2 United States Department of Agriculture (USDA), Agricultural Research Service, University of Illinois, 1101 W. Peabody Drive, Urbana, IL 61801, USA; E-Mail: ghartman@illinois.edu

**Keywords:** soybean, hairy roots, transformation, phytoalexins, resveratrol, pterostilbene, glyceollins

## Abstract

Soybean hairy roots transformed with the *resveratrol synthase* and *resveratrol oxymethyl transferase* genes driven by constitutive *Arabidopsis* actin and CsVMV promoters were characterized. Transformed hairy roots accumulated glycoside conjugates of the stilbenic compound resveratrol and the related compound pterostilbene, which are normally not synthesized by soybean plants. Expression of the non-native stilbenic phytoalexin synthesis in soybean hairy roots increased their resistance to the soybean pathogen *Rhizoctonia solani*. The expression of the *AhRS3* gene resulted in 20% to 50% decreased root necrosis compared to that of untransformed hairy roots. The expression of two genes, the *AhRS3* and *ROMT*, required for pterostilbene synthesis in soybean, resulted in significantly lower root necrosis (ranging from 0% to 7%) in transgenic roots than in untransformed hairy roots that had about 84% necrosis. Overexpression of the soybean *prenyltransferase (dimethylallyltransferase) G4DT* gene in soybean hairy roots increased.

## 1. Introduction

Diseases and pests can keep soybean grain producers from achieving maximum productivity. Host plant resistance is an economical and sustainable disease and pest management option. There is a strong demand for soybean cultivars with improved pest and disease resistance. Progress achieved during the last decade in studies of plant defense mechanisms indicates that one of the important overall transgenic approaches to combat pathogens could be over-expression of genes that produce proteins (pathogenesis-related proteins), involved in the biosynthesis of compounds (phytoalexins or phytoanticipins) that are toxic to pathogens or inhibit their growth and development [1].

Synthesis of phytoalexins or phytoanticipins is considered to be an important part of the plant innate immune response to a variety of pathogens [2,3,4,5,6,7,8,9]. Induction of synthesis of the pterocarpan phytoalexin glyceollin in soybean plants during invasion by pathogens and pests, and by abiotic stresses, has been extensively reviewed by [9]. It was also reported that genetic modification of various plant species capacity to produce phytoalexins affects the transgenic plant resistance to pathogens [9,10,11,12,13,14,15,16,17,18]. Our previous studies showed that transgenic modulation of soybean plant potential to accumulate glyceollin in response to pathogen attacks increased soybean disease resistance, with higher resistance found in plants with elevated glyceollin synthesis [16,19,20,21,22,23].

Expression of the non-native phytoalexin synthesis in crop plants could increase plant immunity and resistance to pests and diseases because host pathogens may have low capability to detoxify non-native phytoalexins, which reduce the rate of colonization until other parts of innate plant defense are activated to maximum levels, such as the production of antimicrobial reactive oxygen species.

Stilbenic compounds with a broad spectrum of biological activities recently generated an interest as nutraceuticals with health promoting effects in humans, such as cardioprotection, anti-inflamatory, neuroprotective and anticancer effects [24,25], and also as antibiotics protecting plants from various microorganisms, nematodes, or herbivores [14,15,18]. Stilbenes occur in plants of several families, such as Cyperaceae, Dipterocarpaceae, Gnetaceae, Fabaceae, Leguminoseae, Pinaceae, Poaceae, and Vitaceae, and are synthesized from the phenylpropanoid substrates that are present in all higher plants and involves the activity of stilbene synthase or resveratrol synthase (STS) [9]. Transformation of different plant species with the *STS* genes was successfully carried out beginning with introduction of the *STS* gene from peanut into tobacco, which resulted in resveratrol synthesis following induction with UV-light [26]. Since then, the *STS* genes have been expressed in tomato [27], barley and wheat [28], alfalfa [29], and grapevine [30]. The expression of the *STS* genes in these studies led to increased STS activity and accumulation of resveratrol glycoside conjugates in transgenic plants and, importantly, resulted in increased plant resistance to fungal pathogens [31]. Thus, transgenic expression of stilbene synthase from grape into tobacco, tomato, and alfalfa resulted in accumulation of conjugates of the non-native phytoalexin resveratrol, and correspondingly, increased resistance to *Botrytis cinerea* [10], to *Phytophthora infestans* [27] and to *Phoma medicaginis* [29]. The combined expression of the *STS* and *resveratrol oxymethyl transferase (ROMT*) genes in transgenic plants can result in accumulation of conjugates of pterostilbene, a double methylated version of resveratrol, which was reported to have higher fungitoxicity compared to resveratrol [32,33,34]. The stable expression of these two genes in the model plants tobacco and *Arabidopsis* resulted in accumulation of pterostilbene conjugates in both species [35,36]. However, we are unaware of any report attributing induction of resveratrol and pterostilbene synthesis in soybean and their effects on plant disease resistance. We have previously found that resveratrol and pterostilbene strongly suppressed the growth of important soybean fungi when present in growth medium containing the compounds [34]. In this paper we provide molecular and biochemical characteristics of soybean hairy roots expressing *resveratrol synthase* and *resveratrol oxymethyl transferase* under control of constitutive promoters which are capable of producing different levels of stilbenic compounds that are normally not synthesized by soybean, and describe the effects of the non-native phytoalexin expression on severity of infection caused by the soybean generalist fungus, *Rhizoctonia solani*. We also describe how overexpression of the *prenyltransferase (dimethylallyltransferase) G4DT* gene, responsible for the key prenylation reaction in the glyceollin synthesis [37], affects the capacity of hairy roots to accumulate native phytoalexin glyceollin in response to fungal infection.

## 2. Results and Discussion

### 2.1. Expression of Non-Native Stilbenoid Phytoalexins in Soybean Hairy Roots

We produced over 50 independent hairy root lines as a model system for functional gene analysis which express either the peanut resveratrol synthase 3 (*AhRS3*) gene or two genes: *AhRS3* and the resveratrol o-methyltransferase (*ROMT)* gene from *Vitis vinifera*, for synthesis of resveratrol and/or pterostilbene, respectively. These genes mediate the synthesis of the non-native stilbenic compounds that are not normally present in soybean plants.

**Figure 1:**
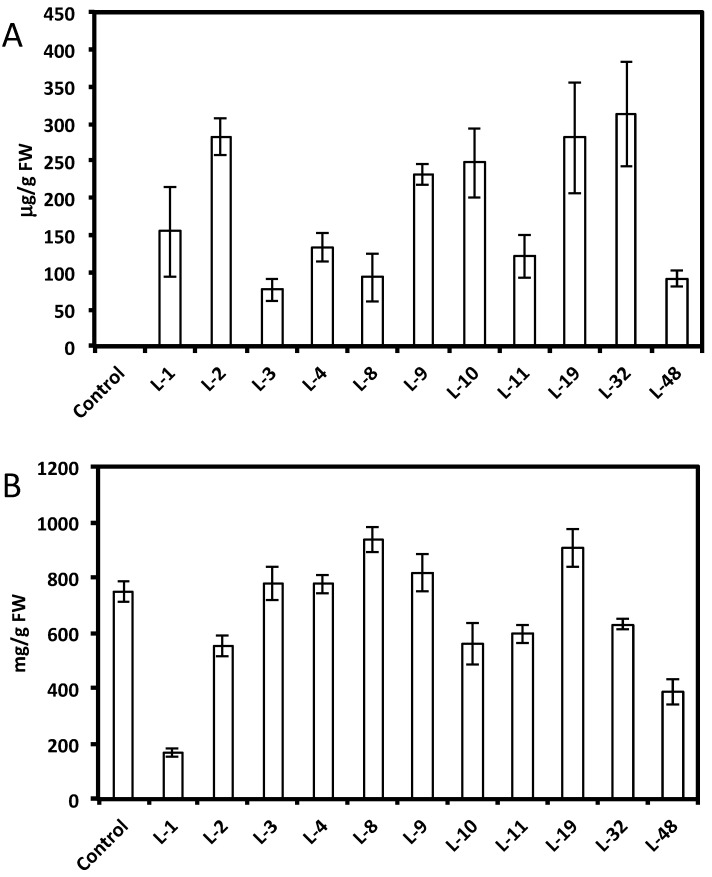
Concentrations of resveratrol (**A**) and isoflavones (**B**) in different soybean hairy root lines derived from soybean cultivar Spencer transformed with the *AhRS3* gene driven by the CsVMV promoter compared with untransformed Spencer hairy roots (control).

**Figure 2:**
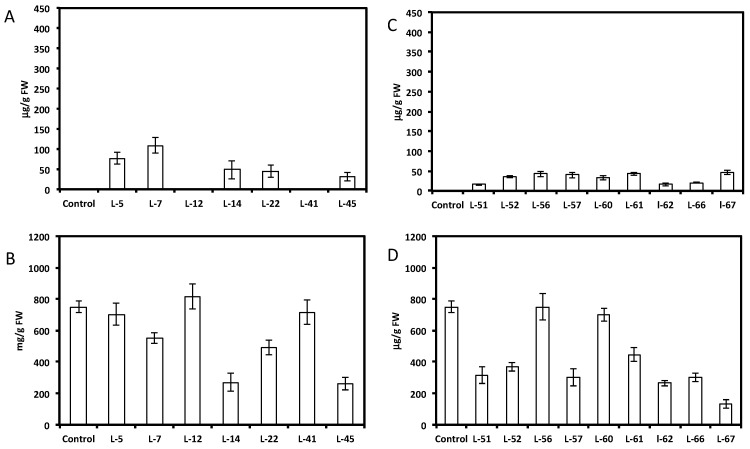
Concentrations of resveratrol (**A** and **C**) and isoflavones (**B** and **D**) in soybean hairy root lines derived from soybean cultivar Spencer transformed with either the *AhRS3* and the *ROMT* genes both driven by the CsVMV promoters (A and B) or with the *AhRS3* driven by the *Arabidopsis* actin promoter and the *ROMT* gene driven by the CsVMV promoter (C and D). The control is the untransformed hairy roots.

**Figure 3:**
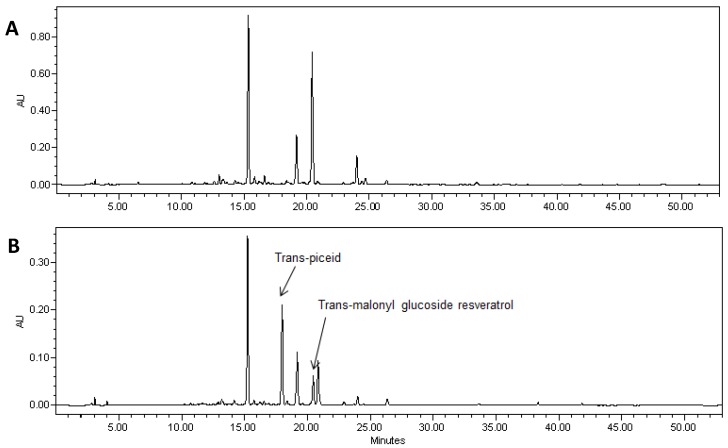
HPLC chromatogram of extracts of soybean hairy roots derived from soybean cultivar Spencer untransformed (**A**) and Spencer transformed with the *AhRS3* gene driven by the CsVMV promoter (**B**). Identified peaks are trans-resveratrol malonyl glucoside, (RT 17.5 min) and trans-piceid (trans-resveratrol glucoside, RT 21.1 min at UV detection (λ = 306 nm).

HPLC analysis showed a wide range of resveratrol concentrations (10–350 μg per gram of fresh root tissue) in transformed root tissues and we did not find resveratrol in the control untransformed hairy roots (Figure 1A and Figure 2A,C). Resveratrol was found in transformed hairy root tissues as glucosyl (trans-piceid) and malonylglucosyl conjugates (Figure 3). The structures of these compounds were confirmed by LC-MS. Trans-piceid gave a molecular ion [M−H]^−^ at *m/z* 389, MS-MS gave the fragment at *m/z* 227, which corresponded to the loss of glucose residue, [M−Glu−H]^−^. Malonyl glucoside resveratrol gave low intensity ion [M−H]^−^ at *m/z* 475 and high intensity ion at *m*/*z* 431, which corresponded to the loss of CO_2_ from malonyl glucoside. Ms-Ms of this ion gave a fragment at *m/z* 227. Concentration of isoflavones in these lines accumulating resveratrol conjugates varied between 100–900 µg/g FW and did not correlate with resveratrol levels (Figure 1B and Figure 2B,D). Generally, higher levels of resveratrol (75–350 µg/g FW) were found in hairy root lines transformed with the *AhRS3* gene only; resveratrol concentrations varied within 30–110 µg/g FW range in roots expressing the *AhRS3* gene under CsVMV promoter and the *ROMT* gene and less than 50 µg/g FW were present in lines expressing the *AhRS3* gene driven by the actin promoter and the *ROMT* gene (these latter hairy roots were produced from seeds of our transgenic plants previously transformed with the *AhRS3* gene under *Arabidopsis* actin promoter after treatment with *A. rhizogenes* carrying the *ROMT* gene under the CsVMV promoter) (Figure 1A and Figure 2A,C).

**Figure 4:**
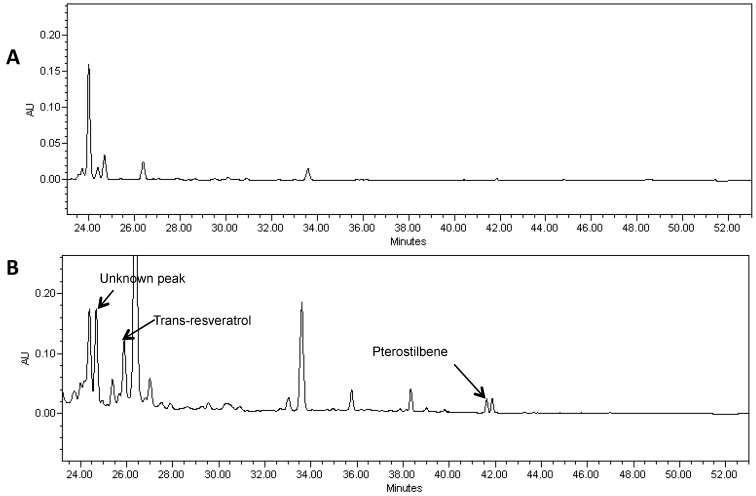
A portion of the HPLC chromatogram of a methanol extract of hairy roots derived from soybean cultivar Spencer (**A**) and Spencer transformed with the *AhRS3* and the *ROMT* genes, both driven by the CsVMV promoters (**B**) and treated with β-glucosidase showing the presence of resveratrol (R_T_ 25.9 min), pterostilbene (R_T_ 41.0 min) and unknown peak (R_T_ 24.3 min) detected only in lines transformed with both genes at UV detection (λ = 306 nm).

Pterostilbene was found in soybean hairy roots that were transformed with the two genes required for pterostilbene synthesis (Figure 4). LC-MS of extracts showed a peak of molecular ion at *m/z* 255 [M−H]^−^ with fragment at *m/z* 241 in MS-MS after loss of the methyl group. It was identical to the commercial standard of pterostilbene. Pterostilbene levels in soybean hairy roots ranged from 5 to 8 µg/g FW.

HPLC analysis also showed a peak with R_T_ of 24.7 min in hairy root lines transformed with both the *AhRS3* and *ROMT* genes and this peak may correspond to a yet non-identified stilbene conjugate (Figure 4—unknown peak).

Several Spencer hairy root lines were selected for further biochemical and molecular analysis: lines 32 and 48 transformed with the *AhRS3* gene under CsVMV promoter; lines 14 and 45 transformed with the *AhRS3* and *ROMT* genes, both driven by CsVMV promoters; and lines 56 and 67 transformed with two genes driven by different promoters, the *AhRS3* gene—under the actin promoter and the *ROMT* gene—under the CsVMV promoter.

**Figure 5:**
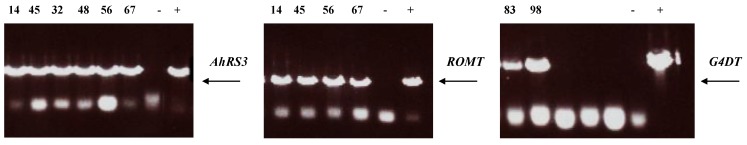
PCR analysis of transgenic hairy root lines derived from soybean cultivar Spencer: Lines 14 and 45 were transformed with the *AhRS3* and the *ROMT* genes both driven by the CsVMV promoters; Lines 56 and 67 were transformed with the *AhRS3* gene driven by the *Arabidopsis* actin promoter and the *ROMT* gene driven by the CsVMV promoter; Lines 32 and 48 were transformed with the *AhRS3* gene driven by the CsVMV promoter; Lines 83 and 98 were transformed with the *G4DT* gene driven by the CsVMV promoter. − negative control, Spencer hairy root, transformed with K599 strain without vector + positive control, plasmid.

**Figure 6:**
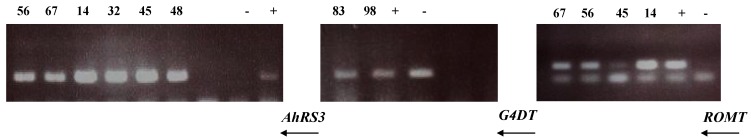
RT-PCR analysis of hairy roots lines derived from soybean cultivar Spencer: Lines 14 and 45 were transformed with the *AhRS3* and the *ROMT* genes, both driven by the CsVMV promoter; Lines 56 and 67 were transformed with the *AhRS3* gene driven by the *Arabidopsis* actin promoter and the *ROMT* gene driven by the CsVMV promoter; Lines 32 and 48 were transformed with the *AhRS3* gene driven by the CsVMV promoter; Lines 83 and 98 were transformed with the *G4DT* gene driven by the CsVMV promoter; − negative control, Spencer hairy root, transformed with K599 strain without vector + positive control, plasmid.

**Figure 7:**
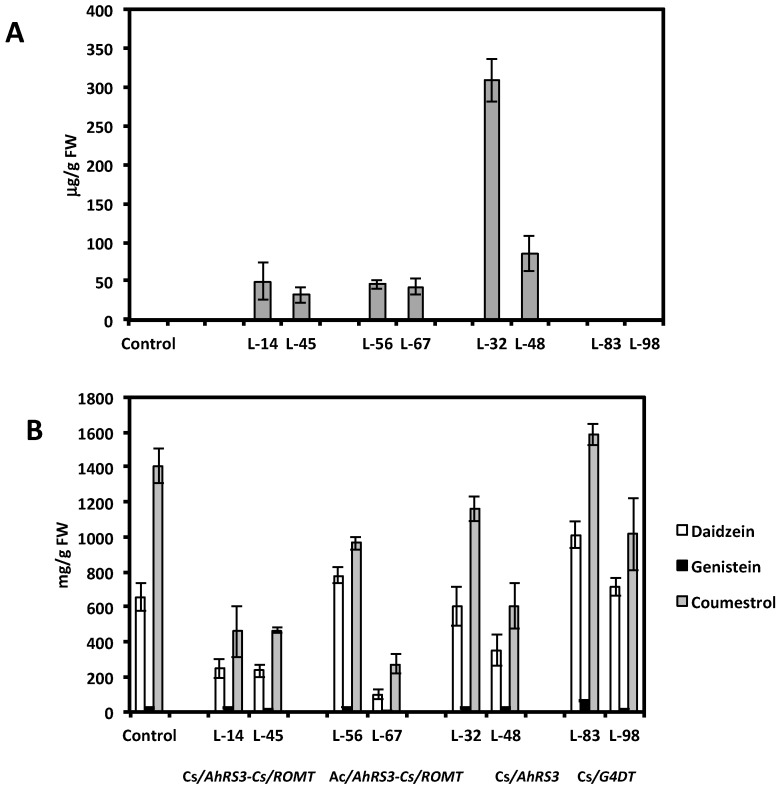
Resveratrol (A) and isoflavones and coumestrol (B) in selected transgenic hairy root lines derived from soybean cultivar Spencer: Lines 14 and 45 were transformed with the *AhRS3* and the *ROMT* genes, both driven by the CsVMV promoters; Lines 56, and 67 were transformed with the *AhRS3* gene driven by the *Arabidopsis* actin promoter and the *ROMT* gene driven by the CsVMV promoter; Lines 32 and 48 were transformed with the *AhRS3* gene driven by the CsVMV promoter; Lines 83 and 98 are transformed with the *G4DT* gene driven by the CsVMV promoter. The control is the untransformed hairy roots.

Presence of transgenes in these selected lines was confirmed by PCR (Figure 5) and the transgene expression was confirmed by RT-PCR (Figure 6) as well as by the presence of stilbenic compounds, the transgene products, which are normally not synthesized in soybean plants (Figure 1, Figure 2, Figure 3, Figure 4 and Figure 7).

**Figure 8:**
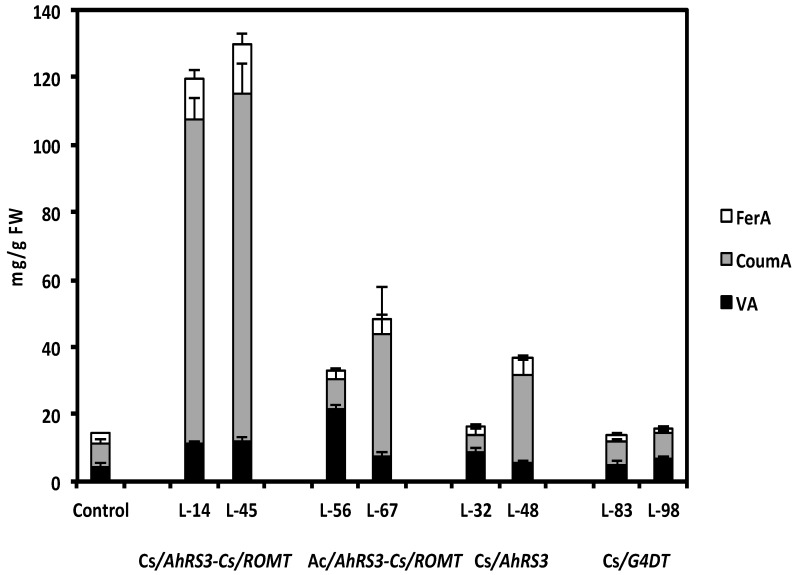
Soluble phenolic acids in selected transgenic hairy root lines after β-glucosidase hydrolysis: Cs—CsVMV promoter, Ac—actin promoter; VA—vanillic acid, CoumA—coumaric FerA—ferulic acid; Lines 14 and 45 were transformed with the *AhRS3* and the *ROMT* genes both driven by the CsVMV promoter; Lines 56 and 67 were transformed with the *AhRS3* gene driven by the *Arabidopsis* actin promoter and the *ROMT* gene driven by the CsVMV promoter; Lines 32 and 48 were transformed with the *AhRS3* gene driven by the CsVMV promoter; Lines 83 and 98 were transformed with the *G4DT* gene driven by the CsVMV promoter. The control is the untransformed hairy roots.

As stated above, concentrations of non-native resveratrol in lines expressing both the *AhRS3* and *ROMT* genes accumulated pterostilbene, and presumably an unidentified stilbenic conjugate, were much lower than in lines transformed with the *AhRS3* gene only. Hairy root lines transformed with both the *AhRS3* and *ROMT* genes also contained markedly reduced isoflavone concentrations compared to untransformed lines, with the exception of line 56 (Figure 7). These lines had elevated concentration of soluble phenolic acids, especially coumaric acid (up to 5 times compared to hairy root lines expressing only the *AhRS3* gene or untransformed lines, see Figure 8). Phenolic acids, detected in these samples, were present as conjugates with glucose, asparagine and aspartate. HPLC chromatograms of hairy root lines transformed with two genes had peaks which were not detected in the untransformed line or in the line transformed with the *AhRS3* gene only. A peak with R_T_ 11.7 min had maximum UV absorption at 308 nm and gave the pseudomolecular ion [M−H]^−^ at *m/z* 277, which produced in MS-MS fragments at *m/z* 260 [M−H−NH_3_] and 145, which corresponded to the loss of asparagine [M−H−asparagine]^−^ and this compound is assigned as coumaroyl asparagine. We previously detected the accumulation of coumaroyl asparagine in our soybean transgenic lines with suppressed chalcone synthase, which accumulated increased concentrations of phenolic acids [20]. Another additional peak (R_T_ 13.9 min, λ_max_ = 307.5 nm) gave a pseudomolecular ion at *m/z* 278 fragmented in MS-MS to the ions at *m/z* 260 and 163 and we assigned its structure as 4-O-aspartate [20].

**Figure 9:**
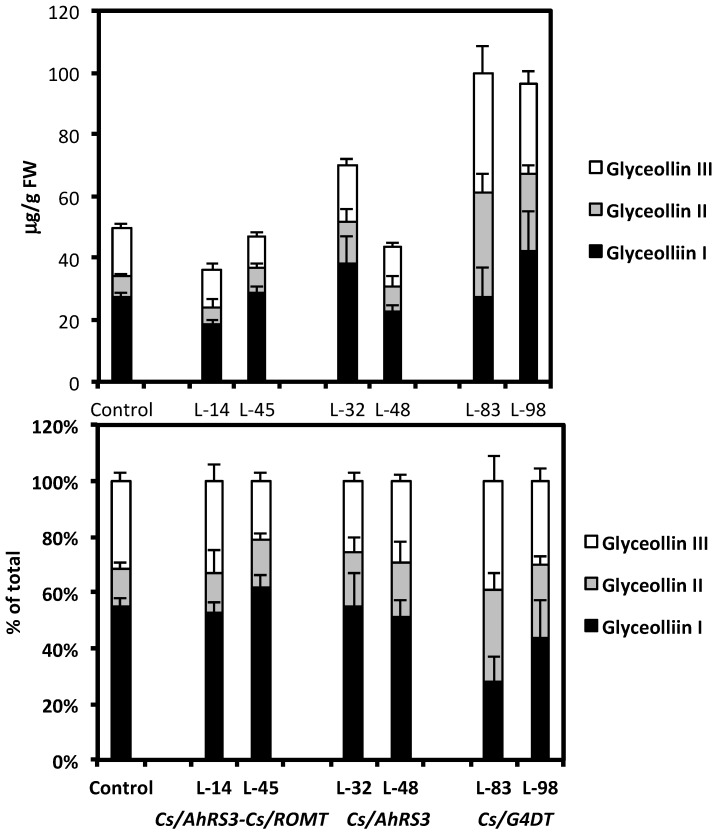
Concentrations (**A**) and proportions (**B**) of glyceollins in hairy roots derived from soybean cultivar Spencer 72 h after inoculation with *Rhizoctonia solani.* Lines 14 and 45 were transformed with the *AhRS3* and the *ROMT* genes, both driven by the CsVMV promoter; Lines 32 and 48 were transformed with the *AhRS3* gene driven by the CsVMV promoter; Lines 83 and 98 were transformed with the *G4DT* gene driven by the CsVMV promoter. The control is the untransformed hairy roots.

We carried out a series of experiments testing the effects of the non-native phytoalexin expression in hairy roots on root necrosis caused by the soybean pathogen *R. solani*. The expression of the *AhRS3* gene resulted in significantly less necrosis in hairy roots that accumulated resveratrol conjugates; it was decreased to about 20%–50% of that of the untransformed hairy roots (Table 1). The expression of two genes, the *AhRS3* and *ROMT*, required for pterostilbene synthesis in soybean, resulted in significantly lower root necrosis (ranging from 0% to 7%) in transgenic roots than in untransformed hairy roots that had about 84% necrosis (Table 1). These results are in agreement with our previous results [34] that indicated a significant inhibition of fungal growth *in vitro* by resveratrol and pterostilbene, with pterostilbene having high fungicidal activity at a lower level (25 µg/mL) than resveratrol (100 µg/mL). Interestingly, levels of native phytoalexin glyceollin accumulated in roots in response to *R. solani* inoculation and the ratio of glyceollins I, II and III were not different between untransformed control roots and hairy roots accumulating stilbenic compounds (Figure 9A, B). This result indicated that synthesis of non-native phytoalexins did not negatively affect accumulation of glyceollin.

**Table 1:** Summary of percent necrosis of hairy root lines derived from soybean cultivar Spencer inoculated with *Rhizoctonia solani* AG4 4 days post-inoculation.

Construct	Line	Percent Root Necrosis^1^	
None	Control	83.7	a
CsVMV/AhRS3	32	42.8	b
CsVMV/G4DT	98	40.5	bc
CsVMV/G4DT	83	39.0	bc
CsVMV/AhRS3	48	18.6	cd
CsVMV/AhRS3& CsVMV/ROMT	22	6.7	de
CsVMV/AhRS3& CsVMV/ROMT	45	6.5	de
CsVMV/AhRS3& CsVMV/ROMT	14	2.8	e
Actin/AhRS3& CsVMV/ROMT	67	1.8	de
Actin/AhRS3& CsVMV/ROMT	56	0.0	e

^1^ Least squares means were estimated from three-six repeated tests with four replications in each test. Common letters indicated means not significantly different (*P* > 0.05) from each other.

Results of these tests described above demonstrate that molecular engineering of soybean to enable plants to synthesize non-native phytoalexins has high potential to increase broad spectrum and durable innate immunity if resistance is similarly increased against other soybean pathogens as was found against *R. solani* in this study and can withstand potential pathogen evolutionary responses to the resistance over time. This approach for improving innate soybean defense against diseases and pests through genetic engineering is novel and to the best of our knowledge, is not being used in the soybean seed industry.

### 2.2. Genetic Modulation of the Native Phytoalexin Glyceollin Synthesis in Soybean Hairy Roots

**Figure 10:**
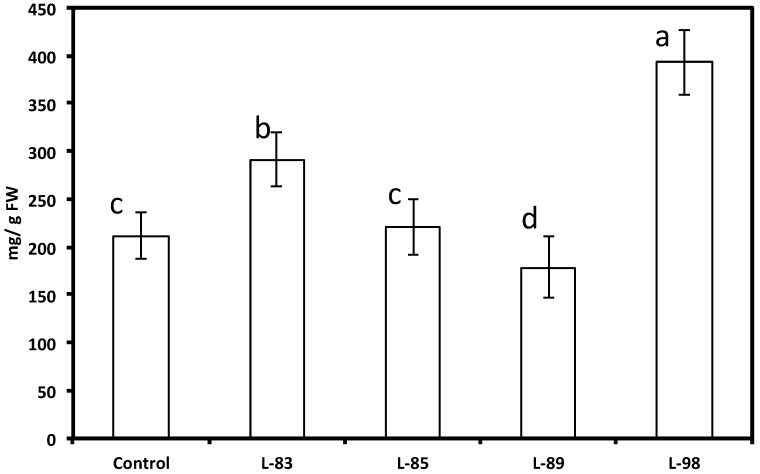
Levels of glyceollins in soybean hairy root lines derived from the soybean cultivar Spencer transformed with the *G4DT* gene 72 h after treatment with HgCl_2_. The control is the untransformed hairy roots. Levels not connected by same letter are significantly different.

With the attempt to increase the capacity of soybean tissues to produce the native soybean phytoalexin glyceollin, we generated new hairy root lines expressing the soybean pterocarpan 4-dimethylallyltransferase (*G4DT)* gene, which controls a key reaction in glyceollin biosynthesis [37]. When several independent hairy root lines derived from soybean cultivar Spencer expressing the *G4DT* gene were subjected to abiotic stress (mercuric chloride), higher levels of glyceollins (up to over 2-fold) accumulated at significantly (*p* < 0.05) higher levels in transformed lines 83 and 98 compared to the untransformed hairy root line in response to this treatment, although induction of glyceollin at some transformed lines was at a similar level (line 85) or significantly lower (line 89) than untransformed roots (Figure 10). The presence of this *G4DT* transgene in hairy roots and its expression was confirmed by PCR and PT-PCR, respectively (Figure 5 and Figure 6, lines 83 and 98). There were no differences in daidzein, genistein and coumestrol concentrations, as well as in concentration and ratio of soluble phenolic acids between these transformed lines and untransformed lines (Figure 7 and Figure 8). When roots of lines 83 and 98 were tested for their response to *R. solani* infection significantly less (about 50%) root necrosis caused by this pathogen was found in these lines compared with the untransformed infected roots (Table 1). Importantly, higher glyceollin accumulation occurred in root tissues of transformed lines 83 and 98 and proportion of glyceollin II was higher in these lines compared to untransformed hairy roots (Figure 9). These results are consistent with our previous results indicating that soybean innate resistance inversely correlates with plant capacity to rapidly induce higher glyceollin synthesis [16,19,20,21,22,23,38]. Therefore, the *G4DT* transgene appears to be a good candidate transgene to move ahead with plant transformation aimed at enhancing plant innate resistance.

Thus, soybean resistance to fungal infection is in part a consequence of the plant’s ability to develop basal defense responses involving phytoalexin production/accumulation and our data reported here reinforce the role played by native and non-native phytoalexins in plant defense. Our results indicated that transgenic modulation of soybean innate resistance, producing a greater accumulation of glyceollin in response to general pathogen invasion, as well as synthesis of stilbenic compounds could help reduce the effects of disease on soybean grain yields and help stabilize soybean production in the presence of diseases. While the pathways studied in the manuscript were constitutively expressed to determine the impact of phytoalexins on soybean pathogens, measure the capacity of soybean tissues to synthesize them, and study the biochemistry of their accumulation, inducible transgenic expression will likely be more sustainable in practice due to less overall demand on plant resources than constitutive expression.

## 3. Experimental

### 3.1. Genetic Modification of Soybean Hairy Roots

Genetic transformation of soybean hairy roots was carried out using the efficient *Agrobacterium*-mediated introduction of genes as previously described [20]. We have used the following three transgenes: 1.5 kb *AhRS3*, peanut *resveratrol synthase* [39] (gene bank accession number AF227963, Celtek Bioscience, Nashville, TN, USA), 1.074 kb *resveratrol o-methyltransferase*, *ROMT* cDNA from *Vitis vinifera* [40] (accession number FM178870.1, Celtek Bioscience), and 1.242 kb soybean cDNA *pterocarpan 4-dimethylallyltransferase*, *G4DT* [37] (accession number AB434690.1, Celtek Bioscience). These transgenes were inserted in the BamHI site in the pILTAB-357 vector for *Agrobacterium rhizogenes* (K599)-mediated transformation of hairy roots [20] and were driven by the CsVMV promoter. To generate hairy root cultures, soybean cotyledons of the cultivar Spencer were treated either with *Agrobacterium* transformed with CsVMV/*AhRS3* or with CsVMV/*G4DT* plasmids or with a mixture (in equal proportion) of *Agrobacterium* cultures transformed with either *AhRS3* or *ROMT* genes driven by the CsVMV promoter. We also treated seeds of transgenic soybean plants previously transformed in our laboratory by biolistic delivery of the *AhRS3* gene under *Arabidopsis* actin promoter in the pAPCH-7 plasmid (gift from Dr. R. Meagher, Department of Genetics, University of Georgia, Athens, GA, USA) with *A. rhizogenes* carrying the *ROMT* gene under control of the CsVMV promoter (lines 56 and 67).

### 3.2. Molecular Analysis

Genomic DNA was extracted from hairy roots using the method of Dellaporta [41] and was used in PCR with primers: 5'*AhRS3*-: ATG GTG TCT GTG AGT GGA ATT CGC AAT GTT, 3'RT-*AhRS3*: AGA TAT ACC CAA AGG ATC AAA to amplify the 1,100 bp fragment of *AhRS3* gene; primers from the CsVMV promoter: 5'AGG ATA CAA CTT CAG AGA and 3'*ROMT*-BamH: GGA TCC TCA AGG ATA AAC CTC AAT GAG GGA CCT CAA to amplify the 1,074 bp of *ROMT* gene; primers from CsVMV promoter (as above) and 3'*G4DT*: TCA TCT AAT TAA TGC CAT GAG AAA GAA CCC to amplify 1242 bp of *G4DT.* The presence of transgenes in root tissues was confirmed by the PCR reaction using Tag DNA polymerase (New England Biolabs, Ipswich, MA, USA) with denaturation at 95 °C-1 min, annealing at 48 °C-40 s and extension at 72 °C-90 s, 35 cycles. “RNeasy Plant Mini Kit” (Qiagen, Valencia, CA, USA) was used for extraction of total RNA from root tissues. DNA was digested with DNase I, using the Ambion@RNA Life Technologies TURBO DNA-free kit (Carlsbad, CA, USA). cDNA was synthesized by reverse transcriptase reaction using oligo (dT) primer and reverse transcriptase enzyme, Promega GoScript kit, (Madison, WI, USA) for positive control or excluding reverse transcriptase enzyme in negative control for genomic DNA contamination. To amplify *AhRS3* transcript from cDNA we used the same primers as for genomic DNA detection. To amplify the 110 bp fragment of the *ROMT* transcript, we used the following primers: RT-*ROMT*-5': TGC CTC TAG GCT CCT TCT AA and RT-*ROMT* -3': TTT GAA ACC AAG CAC TCA GA. To amplify the 570 bp of *G4DT* fragment we used the following primers: 5'CsVMV: AGG ATA CAA CTT CAG AGA and 3'-*G4DT*:GTC CAG ATG CCA TTG GAA GAT GTGG.

### 3.3. Analysis of Phenylpropanoids in Soybean Tissue

Soybean samples frozen in liquid nitrogen were freeze-dried and extracted with 80% methanol [16,19,20,38,42]. Methanol was removed on a rotatory evaporator and the residue was re-suspended in 0.1 M acetate buffer, pH 5.0 and treated with β-glucosidase from almonds (Sigma-Aldrich, St. Louis, MO, USA) overnight at 37 °C. Aglucones were extracted with ethyl acetate, transferred to 80% methanol and analyzed by HPLC using Waters 2690 Separation Module (Waters Corp, Milford, MA, USA) with Prevail C18 column , 250 × 46 mm, 5 µm particle size (Alltech Assoc, Deerfield, IL, USA) with PDA detector Waters 996 and authentic standards as described by [16,19,20,21].

Solvent A was water-acetonitrile-acetic acid (95:5:0.5) and solvent B was acetonitrile-water-acetic acid (95:5:0.5). The flow rate was 1 mL/min. Elution was done with a linear gradient from 5% to 20% B in 10 min, then to 50% for 30 min and to 95% for 7 min. After that the column was washed with 95% acetonitrile for 3 min and equilibrated at 5% B between runs for 3 min. Total sample to sample time was 58 min. Detection was done by UV absorbance at 306 nm for stilbenes and their derivatives, at 295 nm for phenolic acids, and at 285 nm for glyceollins.

LC-MS analysis of soluble phenolics was carried out in Agilent 1100 LC/MSD Trap XCT Plus (Santa Clara, CA, USA) supplied with column and using the same HPLC conditions as above except that flow rate was 500 μL/min. Ion source was ESI, negative mode, nebulizing gas N_2_, 350 °C, 9 mL/min, capillary voltage 3500 V.

Glyceollins II and III were identified using published UV and LC-MS-MS spectra and quantified respectively to glyceollin I isomer. Cis-resveratrol and other stilbenic compounds were identified by UV spectrum and LC-MS. Levels of accumulated glyceollins in transformed and untransformed hairy root lines were analyzed using JMP 11 (SAS Institute, Cary, NC, USA, 2014) Fit Y by X Procedure. Means were separated by least significant difference at α = 0.05. Resveratrol, pterostilbene, isoflavones and coumestrol and their derivatives were identified by comparing of retention times and UV spectra with authentic standards.

### 3.4. Evaluation of Fungal Colonization of Hairy Root Cultures

Hairy roots transformed with vectors containing the four constructs and control vector without constructs were evaluated for disease severity caused by an isolate of *Rhizoctonia solani*, anastomosis group 4 (AG4), originally isolated from infected soybean roots in Urbana, Illinois in 2000, and maintained on potato dextrose agar by serial transfer. The tests were set up as completely randomized designs with four replicates. A 2cm long section of root was cut from an actively growing hairy root culture and placed onto a 60 × 15 mm petri dish (Falcon, Franklin Lakes, NJ, USA) filled with antibiotic (100 ppm/L penicillin and 100 ppm/L streptomycin) − treated 1% water agar. A 4 mm plug, cut from the edge of an actively growing 4-day-old culture using a cork borer, was placed mycelium side down onto the middle of the excised root segment. Plates containing root segments were placed into a closed plastic container and covered with a black cloth to keep the roots in the dark. The plates were kept on a bench-top at 24 °C through the duration of the test. Mycelial plugs were removed from the root segments 24 h post-inoculation. Roots were evaluated by visually estimating the percent necrosis 4 days post-inoculation. Each test was repeated 3–6 times. The percent necrosis data was transformed prior to analysis using arc sine square root transformation to correct for non-constant variance among the treatments (hairy root lines). Transformed data were analyzed using the JMP 11 Fit Model Procedure. Variables were the constructs and lines nested within constructs. Means were separated by least significant difference at α = 0.05.

## 4. Conclusions

Results of this work indicated that the expression of transges, *AhRS3* and *ROMT* controlling the biosynthesis of the non-native stilbenes resveratrol and pterostilbene significantly reduced *R. solani* root colonization in transformed soybean hairy roots. In addition, overexpression of the *G4DT* gene in soybean hairy roots resulted in higher accumulation of the native phytoalexin glyceollins in response to biotic and abiotic stresses. These encouraging results validate the approach to use genetic engineering of soybean and other important crop plants to elevate biosynthesis of native and non-native phytoalexins, with the aim to increase broad-spectrum innate host defenses against pathogens and pests.
